# Enhanced upper genital tract pathologies by blocking Tim-3 and PD-L1 signaling pathways in mice intravaginally infected with *Chlamydia muridarum*

**DOI:** 10.1186/1471-2334-11-347

**Published:** 2011-12-14

**Authors:** Bo Peng, Chunxue Lu, Lingli Tang, I-Tien Yeh, Zhimin He, Yimou Wu, Guangming Zhong

**Affiliations:** 1 Department of Microbiology and Immunology, University of Texas Health Science Center at San Antonio, 7703 Floyd Curl Drive, San Antonio, TX 78229, USA; 2 Department of Pathology, University of Texas Health Science Center at San Antonio, 7703 Floyd Curl Drive, San Antonio, TX 78229, USA; 3 Cancer Research Institute, Xiangya School of Medicine, Central South University, 110 Xiangya Rd, Changsha, Hunan 410078, China; 4 Department of Microbiology and Pathology, University of South China, 28 West Changsheng Rd, Hengyang, Hunan 421001, China

**Keywords:** *Chlamydia muridarum*, Oviduct pathology, Tim-3 &, PD-L1 signaling pathways

## Abstract

**Background:**

Although Tim-3 & PD-L1 signaling pathways play important roles in negatively regulating immune responses, their roles in chlamydial infection have not been evaluated.

**Methods:**

Neutralization antibodies targeting Tim-3 and PD-L1 were used to treat mice. Following an intravaginal infection with *C. muridarum *organisms, mice with or without the dual antibody treatment were compared for live chlamydial organism shedding from the lower genital tract and inflammatory pathology in the upper genital tract.

**Results:**

Mice treated with anti-Tim-3 and anti-PD-L1 antibodies displayed a time course of live organism shedding similar to that of mice treated with equivalent amounts of isotype-matched IgG molecules. The combined antibody blocking failed to alter either the lower genital tract cytokine or systemic humoral and cellular adaptive responses to *C. muridarum *infection. However, the antibody blocking significantly enhanced *C. muridarum*-induced pathologies in the upper genital tract, including more significant hydrosalpinx and inflammatory infiltration in uterine horn and oviduct tissues.

**Conclusions:**

The Tim-3 and PD-L1-mediated signaling can significantly reduce pathologies in the upper genital tract without suppressing immunity against chlamydial infection, suggesting that Tim-3 and PD-L1-mediated negative regulation may be manipulated to attenuate tubal pathologies in women persistently infected with *C. trachomatis *organisms.

## Background

*Chlamydia trachomatis *causes the most frequent sexually transmitted bacterial infections [1-3], which, if untreated, can lead to complications characterized with tubal inflammatory damages, including pelvic inflammatory diseases, ectopic pregnancy and infertility [1,4,5]. Although both intracellular replication of *C. trachomatis *organisms and host responses to *C. trachomatis *antigens may significantly contribute to inflammatory pathologies [6-9], the precise pathogenic mechanisms of *C. trachomatis*-induced diseases remain unknown. In addition, there is still no licensed *C. trachomatis *vaccine [10] despite the urgent need and extensive efforts in searching for such a vaccine.

Previous immunological studies, mainly based on a *C. muridarum *intravaginal infection mouse model, have revealed that a Th1-dominant cell-mediated immunity is required for protection against Chlamydia urogenital tract infection [10-12]. It is also hypothesized that excessive and/or prolonged cellular (particularly CD + 8 T cell) responses may contribute to tubal pathologies during chlamydial infection [13,14]. However, how these protective and pathogenic cellular responses are regulated remains unknown. Both Tim-3 (T cell immunoglobulin and mucin domain 3) and PD-1 (Programmed death one) are negative regulators of T cell responses [15,16]. We evaluated the role of these two negative regulatory signaling pathways in chlamydial urogenital infection in the current study.

Tim-3-mediated signal inhibits both CD4+ Th1 and CD8+ T cell responses, which may prevent unintended tissue inflammation [17]. However, inappropriate activation of Tim-3 signals may lead to premature T cell exhaustion, thus, permitting persistent or chronic infection [18-21]. Tim-3 has emerged as a promising therapeutic target to correct abnormal immune function in several autoimmune and chronic inflammatory conditions [22]. PD-1 is an inducible molecule on activated T and B lymphocytes and plays a critical role in controlling lymphocyte activation and maintaining peripheral tolerance [19,23]. PD-L1, the primary regulatory counter-receptor for PD-1 in the peripheral tissues is broadly inducible in various tissues and cell types [23-26]. The interaction between PD-1 and PD-L1 plays a critical role in determining the fate of T-cell activation and tolerance during T-cell priming [23]. Like Tim-3, inappropriate activation of PD-1 signaling can lead to immune suppression and persistent/chronic infection [27]. For example, PD-1-PD-L1 pathway has been shown to impair Th1 immune response in the late stage of infection with *Mycobacterium bovis bacillus Calmette-Guérin*, thereby facilitating the bacterial persistence in the host [28]. Decrease in the exhaustion markers PD-1 and TIM-3 in T cells correlates with reduction of *Mycobacterium tuberculosis *load in the lungs [29]. Thus, blocking PD-1 signaling pathway may prevent persistent infection.

However, Targeting the PD-1-PD-L1 pathway alone does not always result in complete restoration of T cell function [30]. Double blocking with neutralization antibodies against both Tim-3 and PD-L1 has been shown to restore T cell function in both solid tumor-bearing mice [31] and mice chronically infected with viruses [32], leading to controlling tumor growth and viral infection respectively. Thus, in the current study, we used a combined blocking approach to assess the effect of Tim-3 and PD-L1 signaling pathways on Chlamydia infection in a *C. muridarum *intravaginal infection mouse model. We found that the *C. muridarum *organism shedding time course after an intravaginal infection was not altered despite the double blocking. However, the tubal pathology following the *C. muridarum *infection was more severe in mice treated with neutralization antibodies targeting both Tim-3 and PD-L1. These observations suggest that Tim-3 and PD-L1 signaling may play an important role in reducing pathologies in the upper genital tract after chlamydial infection.

## Materials and methods

### Mouse infection, antibody treatment and titration of live organism shedding

*C. muridarum *Nigg strain (also called MoPn) organisms used in the current study were grown in HeLa cells (ATCC, Manassas, VA 20108), purified and titrated as described previously [9]. Female Balb/c mice were purchased at the age of 6 to 8 weeks old from Charles River Laboratories, Inc. (Wilmington, MA). Each mouse was inoculated intravaginally with 2 × 10^4 ^IFUs of live *C. muridarum *organisms as described previously [9]. After infection, the mice were treated with either neutralization antibodies or isotype control IgG for 12 days as following: For the anti-Tim-3 + PD-L1 group, each mouse was injected intraperitoneally (i.p.) with 100 μg of anti-Tim-3 (clone# 8B.2 C12; Rat IgG1, cat#16-5871, eBioscience, San Diego, CA) on days 0 (the same day of infection), 2 and 4, 200 μg of anti-PD-L1 (clone 10 F.9 G2, Rat IgG2b, cat#124309, Biolegend, San Diego, CA) on days 0, 3, 6, 9 and 12. For the control group, each mouse was similarly injected with the equivalent amounts of isotype-matched rat IgGs following the same injection schedule. After the intravaginal infection, vaginal swabs were taken once every 7 days until two consecutive negative detection results were obtained from the same mouse. The chlamydial organisms released from swabs were inoculated onto HeLa cell monolayers in duplicates as described previously [8]. An immunofluorescence assay was used to quantitate live organisms (expressed as inclusion forming units or IFUs) from each swab as described previously [9]. The number of IFUs/swab was converted into log_10 _and the log_10 _IFUs were used to calculate means and standard deviation for each group at each time point.

### Evaluating mouse genital tract tissue pathologies and histological scoring

Mice were sacrificed 60 days after infection and the mouse urogenital tract tissues were isolated. Before the tissues were removed from mice, an *in situ *gross examination was performed for evidence of hydrosalpinx formation and any other abnormalities. The excised tissues were processed for making serial sections. Efforts were made to include cervix, both uterine horns and oviducts as well as lumenal structures of each tissue in each section. The sections were stained with hematoxylin and eosin (H&E) and assessed by a board certified pathologist (I-T.Y) blinded to mouse treatment for severity of inflammation and pathologies based on the modified schemes established previously [8]. The uterine horns and oviducts were scored separately. Scoring for dilatation of uterine horn or fallopian tube: 0, no significant dilatation; 1, mild dilatation of a single cross section; 2, one to three dilated cross sections; 3, more than three dilated cross sections; and 4, confluent pronounced dilation. Scoring for inflammatory cell infiltrates (at the chronic stage of infection, the infiltrates mainly contain mononuclear cells): 0, no significant infiltration; 1, infiltration at a single focus; 2, infiltration at two to four foci; 3, infiltration at more than four foci; and 4, confluent infiltration. Scores assigned to individual mice were calculated into means ± standard errors for each group of animals.

### Immunofluorescence assay

For titrating IFUs from mouse swab samples, HeLa cells grown on glass coverslips in 24 well plates were inoculated with serially diluted swab samples and processed for immunofluorescence assay 24 h after infection. The cell samples were labeled with Hoechst (blue, Sigma) for visualizing DNA and a rabbit anti-chlamydial chaperon cofactor antibody (unpublished data) plus a goat anti-rabbit IgG conjugated with Cy2 (green; Jackson ImmunoResearch Laboratories, Inc., West Grove, PA) for visualizing chlamydial inclusions. The immuno-labeled cell samples were quantitated and used for image analysis and acquisition as described previously [33,34]. For titrating anti-*C. muridarum *antibodies in mouse serum samples, the *C. muridarum*-infected HeLa cells were processed and added with serially diluted mouse serum samples. After the primary antibody binding, a goat anti-mouse IgG conjugated with Cy3 (red; Jackson ImmunoResearch Laboratories) was used to visualize the mouse antibody binding to *C. muridarum *organisms. The antibody titer was defined as the highest dilution that still showed positive fluorescence labeling and expressed as log10 dilutions for statistic analyses.

### Enzyme-Linked Immunosorbent Assay (ELISA)

Cytokines from mouse vaginal swabs and mouse splenocyte culture supernatants (after in vitro restimulation) were measured using Bio-plex Pro™ mouse cytokine standard Group I 23-plex (cat# 171I5001, BIO-RAD, Hercules, CA) and Group II 9-plex (cat# 171I6001) kits by following the manufacturer's instruction. Since all 32 cytokine beads are uniquely labeled, they were mixed and used in the same assays. For swab samples, after the *C. muridarum *organism titration, each of the left over swab samples was diluted 1:2 in PBS containing 10% fetal bovine serum. 50 ul of the diluted sample was added to each reaction well containing the 32 cytokine beads (each type of beads is covalently coated with a capture antibody specifically recognizing one cytokine). To prepare the splenocyte samples, splenocytes were harvested from mice 60 days after *C. muridarum *infection and stimulated *in vitro *with UV-inactivated *C. muridarum *organisms (elementary bodies or EBs) or medium alone for 3 days. The culture supernatants were diluted 1:2 and 50 ul were used to react with the 32 cytokine beads. The bead capture antibody-bound cytokines were detected with biotinylated detection antibodies and phycoerythrin fluorescence-conjugated Avidin. The fluorescence-associated with each cytokine was quantitated using a Bio-Plex Luminex system (BIO-RAD). The concentrations of each cytokine were determined based on the standards measured in the same plate and sample dilution factors using Bio-Plex Manager software (BIO-RAD) and expressed as pg/ml. The 32 cytokines analyzed were: IL-1α, IL-1β, IL-2, IL-3, IL-4, IL-5, IL-6, IL-9, IL-10, IL-12 (p40), IL-12 (p70), IL-13, IL-17, Eotaxin, G-CSF, CM-CSF, IFN-γ, KC, MCP-1, MIP-1α, MIP-1β, RANTES, and TNFα, IL-15, IL-18, Basic FGF, LIF, M-CSF, MIG, MIP-2, PDGF-BB and VEGF.

### Statistical analysis

ANOVA Test (http://www.physics.csbsju.edu/stats/anova.html) was performed to analyze the IFU, cytokine & antibody titration data from multiple groups and a two-tailed Student *t *test (Microsoft Excel) to compare two given groups. For qualitative data analyses, the Chi-square or Fisher's Exact tests were used.

## Results

### Blockade of Tim-3 and PD-L1 signaling pathways did not alter live organism shedding following an intravaginal infection with *Chlamydia muridarum*

**Figure 1:**
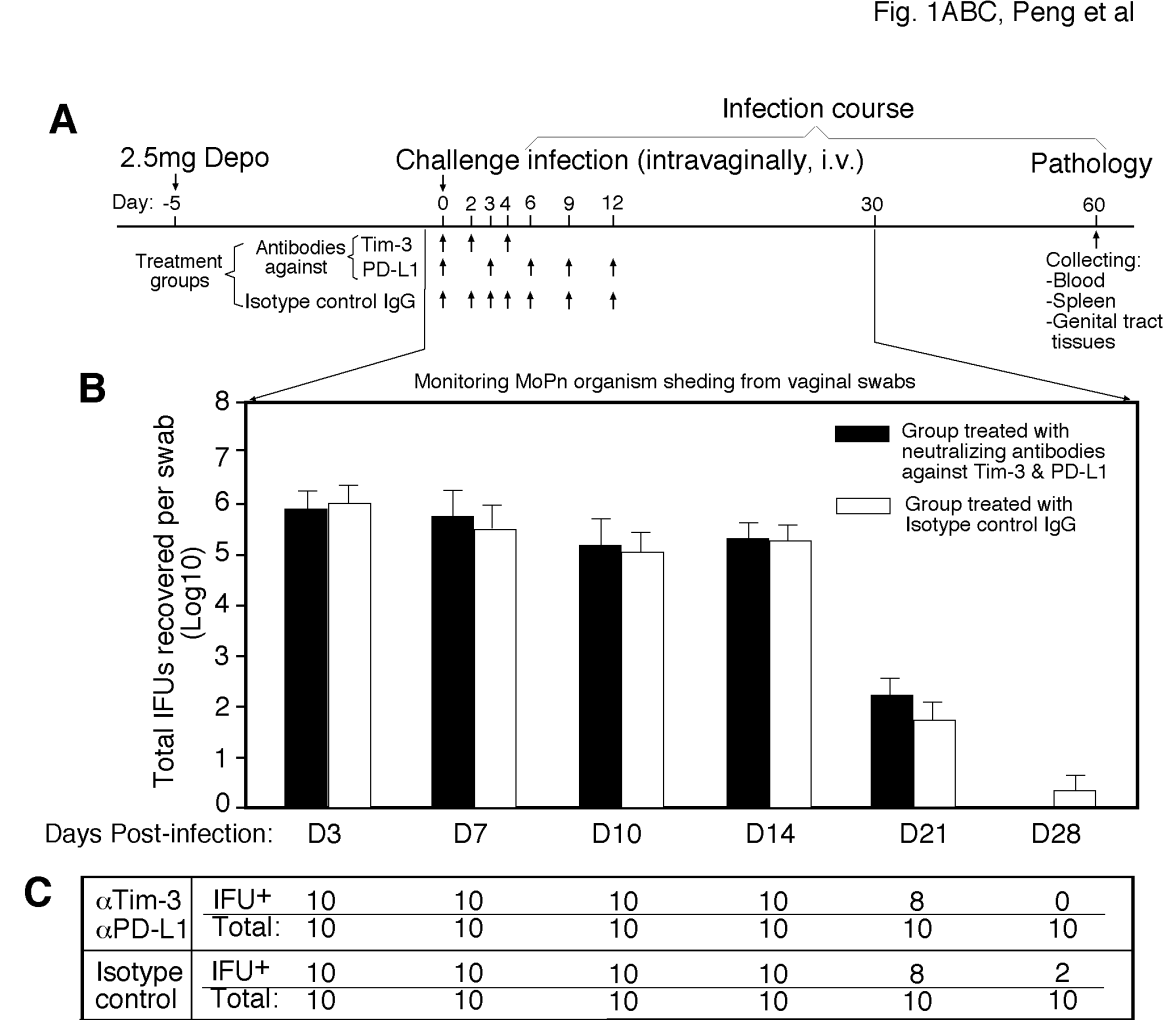
**Effect of targeting the Tim-3 and PD-1 signaling pathways on live organism shedding following chlamydial infection**. (**A**) All mice were intravaginally infected with live *C. muridarum *organisms and vaginal swabs were taken along the infection course as indicated along the horizontal line for monitoring the shedding of live organisms. One group of mice were treated with anti-Tim-3 plus anti-PD-L1 neutralization antibodies while the other group with isotype-matched rat IgGs as indicated. (**B**) The number of live organisms recovered from each swab was expressed as IFUs. After converting into Log_10_, and the log_10 _IFUs were used to calculate mean and SD for each mouse group as displayed along the y-axis and at a given time point along the infection course (X-axis). The log_10 _IFUs along the time course were analyzed with ANOVA, and no significant differences at any time points were found between the control and Tim-3 + PD-L1 groups. (**C**) Number of mice still positively shedding live chlamydial organisms (IFU+) versus the total of mice (total) in each group (antibody treatment group, αTim-3 & αPD-L1 or isotype control group) were listed as function of infection time (horizontally).

Mice treated with a combination of neutralization antibodies against Tim-3 and PD-L1 or isotype-matched control IgG were compared for live organism shedding following an intravaginal infection (Figure 1). A schedule of antibody treatment for 12 days after infection was used (panel A) based on previous studies showing that such a treatment schedule is most effective in inhibiting Tim-3 and PD-L1 signaling in other disease models [31,32]. However, even the double blocking of Tim-3 and PD-L1 signaling did not affect the time course of vaginal shedding of infectious organisms (panels B & C). All mice shed more than 100 thousand live organisms during the first 2 weeks after *C. muridarum *infection. By day 21, 80% mice from both groups each released about 100 live organisms. By day 28, most mice cleared infection. These observations suggest that inhibition of Tim-3 and PD-L1 signaling during the first 2 weeks post infection did not significantly affect host immunity against *C. muridarum *intravaginal infection.

### Inhibition of Tim-3 and PD-L1 signaling pathways significantly enhanced upper genital tract pathologies

**Figure 2:**
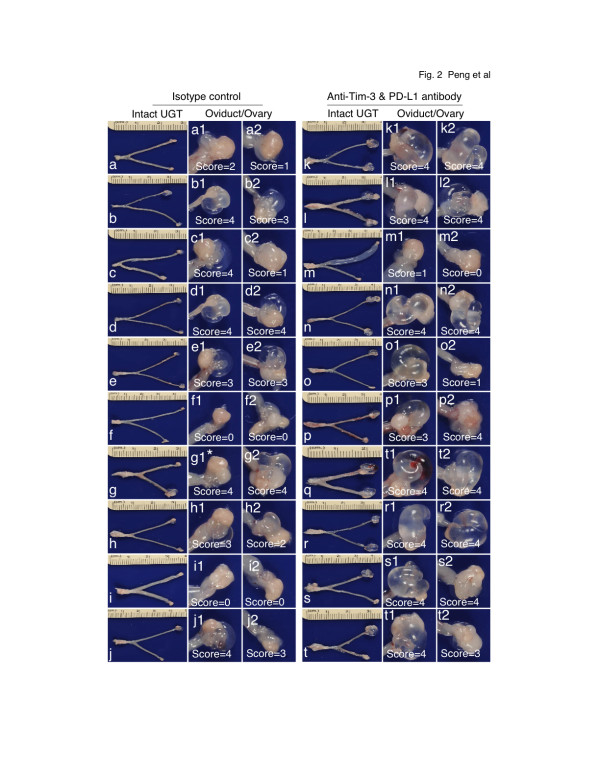
**Effect of targeting the Tim-3 and PD-1 signaling pathways on the development of urogenital tract gross pathology**. Groups of control and anti-Tim-3 + PD-L1 treatment mice were infected with *C. muridarum *as described in Figure1 legend. Sixty days after infection, mice were sacrificed for harvesting genital tissues and the pathology of the mouse genital tissues was evaluated under both naked eyes for gross appearance. The left column in each group (a-j from control and k to t from antibody treatment group) displayed the overall appearance of the entire upper genital tract (UGT), whereas the right two narrow columns were amplified images of the oviduct/ovary portion from both left (for example, marked with a1) and right (a2) sides. Swollen oviducts from either side were marked according to the severity as followings: No hydrosalpinx is assigned a score of zero (0); Hydrosalpinx is only visible after amplification (1); Hydrosalpinx is clearly visible with naked eye but the size is smaller than that of ovary (2); The size of hydrosalpinx is similar to that of ovary (3) or larger than ovary (4). * indicates a swollen oviduct with a severity score of 4 (panel g1) but the bubble was accidentally broken during isolation. The incidence and severity of hydrosalpinx were summarized in Table 1.

**Figure 3:**
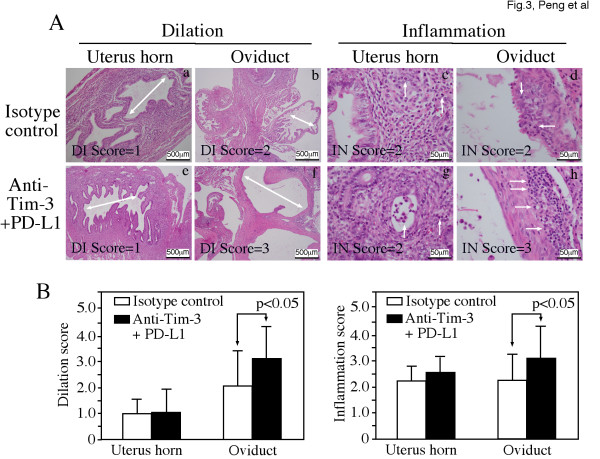
**Effect of targeting the Tim-3 and PD-L1 signaling pathways on upper genital tract inflammatory pathology examined microscopically**. (**A**) After the gross appearance observation, the same tissues were subjected to histological section analyses under microscope (after H&E staining). Representative H&E stained section images covering either uterine horn (panels a, c, e & g) or oviduct (b, d, f & h) regions were presented from each group (a-d from control group while e to h from anti-Tim-3 + PD-L1 treatment group). Each section was scored for both lumenal dilatation (DI, panels a, b, e & f) and inflammatory infiltration (IN, panels c, d g & h). Lumenal dilation was marked with a white line with arrowheads at both ends and while infiltrates marked with white arrows. The semi-quantitation results were presented in **(B)**. Dilation (left plot) and inflammation (right plot) scores (derived from 5 different sections) assigned to individual mice were used to calculate the means and standard errors for each group as shown along the Y-axis. The mouse and tissue groups were indicated either inside the plots (open bar for control while solid bar for antibody treatment groups) or along the X-axis. The scores were compared between different groups using ANOVA followed by the two-tailed Student *t *test. The p values with statistic significance were indicated accordingly.

When the gross pathology of urogenital tract tissues harvested 60 days after infection was compared between the two groups of mice (Figure 2 & Table 1), we found that 8 of the 10 mice from the isotype control group and all 10 mice from the antibody treatment group developed hydrosalpinx although the severity of hydrosalpinx varies a great deal among different oviducts. We developed a scoring system for semi-quantitatively assessing the severity of hydrosalpinx in each oviduct over the years [8]. A total of 14 out of the 20 oviducts from the 10 antibody-treated mice received the highest hydrosalpinx severity score 4 while only 7 oviducts from the isotype control group received the same score (p < 0.05). This observation suggests that blocking Tim-3 & PD-L1 signaling pathways may promote the inflammatory responses in the oviduct and enhance the progression of hydrosalpinx. When H&E-stained mouse urogenital tissues were examined under a microscope, inflammatory infiltrates were significantly higher in oviduct tissues of mice treated with anti-Tim-3 & PD-L1 antibodies (Figure 3). The neutralization antibody-treated mice also displayed more severe oviduct luminal dilatation. Thus, blocking Tim-3 and PD-L1 signaling pathways can lead to exacerbation of tubal inflammatory pathologies.

**Table 1:** Incidence of gross pathologies

Group	Total mice	No of mice			Total oviducts	No of oviducts with hydrosalpinx scores					
				
		None	Uni	Bi		0	1	2	3	4	Total score
Isotype control	10	2	0	8	20	4	2	2	5	7*	49

αTim-3+ αPD-L1	10	0	1	9	20	1	2	0	3	14*	67

*p = 0.028

Mice were sacrificed 60 days after infection and the reproductive tissues were isolated and inspected for gross pathologies (see Figure 2 for images). Number of mice without (none) or with hydrosalpinx on single (Uni) or both (Bi) sides of the reproductive tissues was listed on the left portion of the table. Number of oviducts without (0) or with varying severity degrees (1 to 4) of hydrosalpinx was listed on the right portion of the table. Fisher Exact test was used to compare the differences in incidences of mouse with hydrosalpinx or oviducts with varying degrees of hydrosalpinx between the control and antibody treatment groups. P-vales with statistic differences were marked. * stands for *p *< 0.05.

### The effect of Tim-3 and PD-L1 blockade on genital tract cytokine responses and adaptive immunity to *C. muridarum *infection

**Figure 4:**
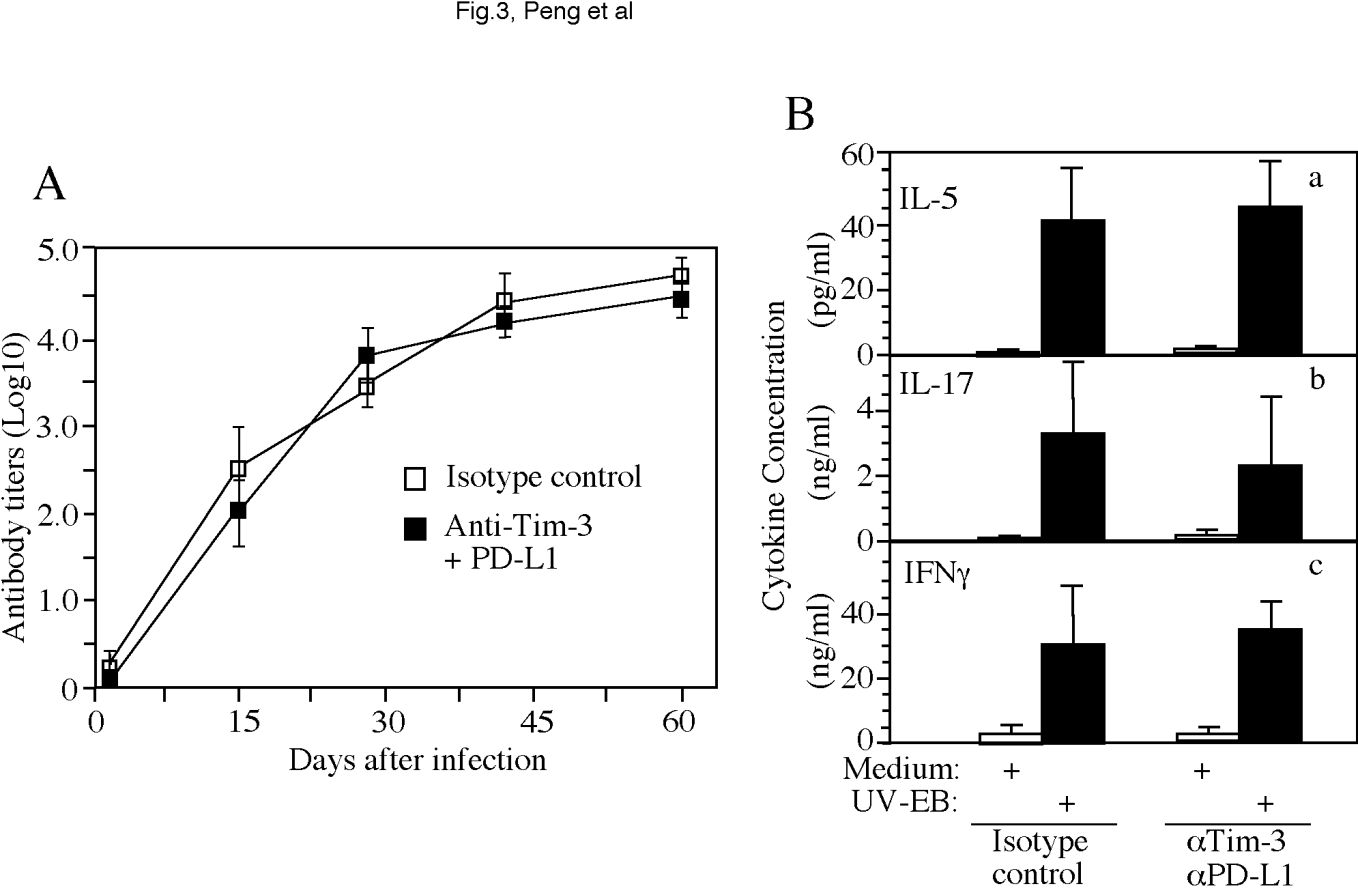
**Effect of targeting the Tim-3 and PD-1 signaling pathways on *C. muridarum*-specific humoral and cellular responses**. (**A**) Serum IgG antibodies from both groups (open squares for control and filled squares for anti-Tim-3 + PD-L1 treatment) were titrated using *C. muridarum*-infected HeLa cells as antigens under an immunofluorescence assay. The Log10 dilution was used to calculate the mean and standard deviation from each group as displayed along the Y-axis and at a given time along the infection time course (X-axis). There was no statistic difference in antibody titers between the two groups at any time points (ANOVA). (**B**) A total of 32 mouse cytokines were measured (using the Bio-Rad Bio-Plex kits) from the supernatants of splenocytes harvested from either control or antibody-treated groups were *in vitro *restimulated with medium alone (open bar) or UV-inactivated *C. muridarum *EBs (solid bar) as indicated along the X-axis. The cytokine concentrations from each group and restimulation condition were calculated in pg or ng/ml as mean and standard deviation as displayed along the Y-axis. Only the cytokines representing Th2 (IL-5, panel a), Th17 (IL-17, b) and Th1 (IFNg, c) were shown and the other 29 cytokines were not shown. There was no statistic difference in any of the 32 cytokine concentrations between the two groups.

To understand the immunological basis of the Tim-3 & PD-L1 blockade-enhanced tubal inflammation, we monitored the cytokine production in the mouse vaginal swab samples using the Bio-Rad Bio-Plex cytokine kits (data not shown). A total of 32 cytokines were measured in swab samples harvested on day 3, 7 & 14 after *C. muridarum *infection. There were no significant differences in any of the cytokines measured between the antibody treatment and control groups (data not shown). We further monitored the adaptive immunity by measuring *C. muridarum*-specific humoral (Figure 4A) and T cell responses (Figure 4B). Both groups of mice produced robust antibody responses and there was no significant difference in antibody titers between the two groups. More importantly, the two groups of mice also displayed similar phenotypes of *C. muridarum*-specific T cell responses and there was no significant difference in the amounts of IL-5 (by Th2 cells), IL-17 (Th17) or IFNg (Th1-like) produced in the supernatants of *C. muridarum*-restimulated splenocytes harvested from the two groups of mice.

## Discussion

Using a combined blocking of Tim-3 and PD-L1, we assessed the role of Tim-3 and PD-L1 signaling pathways in chlamydial infection. Despite the double blockade, the time course of live organism shedding from the lower genital tract after *C. muridarum *infection was not significantly altered. Since the double blocking with neutralization antibodies targeting both Tim-3 and PD-L1 has been shown to effectively suppress both Tim-3 and PD-L1 signaling pathways [31,32], the above result suggests that Tim-3- and PD-1-meditaed negative regulation signaling pathways might not significantly suppress host immunity against chlamydial infection in lower genital tract. This conclusion is consistent with the observations that treatment with anti-Tim3 and PD-L1 neutralizing antibodies did not alter either the lower genital tract local cytokine production or the systemic humoral or cellular responses to *C. muridarum *infection. The fact that the antibody treatment did not affect the balance of Th1 versus Th2 responses may explain the failure of the antibody blocking to impact the lower genital tract infection time course. However, the treatment of mice with anti-Tim-3 and anti-PD-1 neutralization antibodies did significantly enhance pathologies in the upper genital tract, demonstrating that the antibody treatment was effective in blocking of Tim-3 and PD-L1 signaling pathways. More importantly, this observation suggests that Tim-3- and PD-L1-mediated negative regulation signaling pathways may play significant roles in reducing pathogenic responses induced by chlamydial infection in the upper genital tract. Apparently, this negative regulation didn't make the lower genital tract more susceptible to chlamydial infection since removal of the negative regulation did not reduce live chlamydial organism shedding.

Given the multifaceted roles of Tim-3 and PD-L1 signaling pathways, it is unexpected that double blocking of these two pathways did not alter *C. muridarum *infection time course in the lower genital tract. The combined blocking of Tim-3 and PD-L1 has been shown to fully restore the functionality of exhausted T cells expressing both Tim-3 and PD-1 in tumor-bearing animals and chronically infected hosts with viruses [31,32]. Under these chronic pathogenic conditions, the negative regulatory roles of Tim-3 and PD-1 may be heightened or exacerbated. The impact of inhibition of the exacerbated negative regulation can be more sensitively detected immunologically and clinically. However, intravaginal infection with *C. muridarum *is an acute infection. The role of Tim-3 and PD-1 in acute infection is not as well understood as in chronic infection. A recent study has shown that Th1 immune responses at the lower genital tract are regulated by IL-10-producing dendritic cells [35], which may be independent of Tim-3- or PD-1-mediated mechanisms. In addition, it has been demonstrated that interaction of Tim-3 on T cells with its ligand Galectin9 (Gal9) on macrophages may promote clearance of intracellular *Mycobacterium tuberculosis *[36]. Tim3-Gal9 interaction leads to macrophage activation and stimulates bactericidal activity by inducing caspase-1-dependent IL-1β secretion [36]. Thus, Tim-3 may also play a positive role in controlling chlamydial infection. Blocking Tim-3 signaling with neutralizing antibodies remove both the negative and positive roles of Tim-3 in chlamydial infection, resulting in null net effect on the live organism shedding time course. However, since the double antibody blocking treatment affected neither the lower genital tract local cytokine nor the systemic adaptive immune responses to *C. muridarum *infection, it is more likely that the negative regulation mediated by Tim-3 and PD-L1 did not significantly inhibit host immunity during the acute phase of *C. muridarum *in the lower tract.

The double antibody treatment significantly enhanced tubal inflammatory pathology, which is consistent with the overall concept that the negative regulation mediated by Tim-3 and PD-L1 plays an important role in minimizing inflammatory damages caused by excessive and prolonged immune responses [26,37]. What is the cellular basis of the tubal pathology-exacerbating responses negatively regulated by Tim-3 and PD-L1? Although Tim-3 and PD-1 can negatively regulate both CD4+ Th1 and CD8+ T cells, under various chronic infection or tumor-bearing conditions, the frequency of antigen-specific CD8+ T cells positive for both TIM-3 and PD-1 is the highest [16,19,21]. Double blocking with neutralization antibodies to Tim-3 and PD-1 could fully restore the functionality of the exhausted CD8+ T cells. Thus, it is assumed that the enhanced anti-tumor or anti-viral effects restored by the anti-Tim-3 and PD-1 antibodies were due to the removal of negative regulation from CD8+ T cells. Although the cellular basis of inflammatory responses involved in tubal pathology after chlamydial infection is still not clear, some previous studies have correlated CD8+ responses with pathogenic responses in the upper genital tract [13]. Indeed, mice deficient in MHC class I or CD8 displayed normal infection time courses in the lower genital tract [12,38] but developed less severe pathology in the upper genital tract. TNFα produced by CD8+ T cells may contribute to the tubal pathologies [13]. It is possible that Tim-3 and PD-L1 may mainly target antigen-specific CD8+ T cells during chlamydial infection as observed during mycobacterial infection [28].

## Conclusions

Dual blockade of Tim-3 and PD-L1-mediated signaling pathways significantly reduced inflammatory pathologies in the upper genital tract but without affecting immunity in the lower genital tract against chlamydial infection suggests that Tim-3 and PD-L1-mediated negative regulation may be manipulated to attenuate tubal pathologies in women persistently infected with *C. trachomatis *organisms.

## Competing interests

The authors declare that they have no competing interests.

## Authors' contributions

BP & CL carried out most experiments. LT measured cytokines & IY evaluated pathology. ZH, YW & GZ conceived of the study, and participated in its design and drafted the manuscript. All authors read and approved the final manuscript.

## Funding

Support in part by grants (to G. Zhong) from the US National Institutes of Health & Merck.

## Pre-publication history

The pre-publication history for this paper can be accessed here:

http://www.biomedcentral.com/1471-2334/11/347/prepub
